# Coproduction of Enzymes and Beta-Glucan by *Aspergillus oryzae* Using Solid-State Fermentation of Brown Rice

**DOI:** 10.4014/jmb.2105.05005

**Published:** 2021-06-03

**Authors:** Su Bin Ji, Chae Hun Ra

**Affiliations:** Department of Food Science and Biotechnology, College of Engineering, Global K-Food Research Center, Hankyong National University, Anseong 17579, Republic of Korea

**Keywords:** Brown rice, enzyme production, β-glucan, *Aspergillus oryzae*, solid-state fermentation

## Abstract

The effect of medium composition on enzyme and β-glucan production by *Aspergillus oryzae* KCCM 12698 was investigated. Brown rice, rice bran, nitrogen, and ascorbic acid are key components of the synthetic medium used in liquid-state fermentation. To determine the optimal concentrations of these components for enzyme and β-glucan production, we conducted one factor at a time experiments, which showed that the optimal concentrations were 30 g/l brown rice, 30 g/l rice bran, 10 g/l soytone, and 3 g/l ascorbic acid. Pretreatment of brown rice for 60 min prior to inoculation enhanced fungal biomass, while increasing the production of enzymes and β-glucan using solidstate fermentation. Maximum fungal biomass of 0.76 mg/g, amylase (26,551.03 U/g), protease (1,340.50 U/g), and β-glucan at 9.34% (w/w) were obtained during fermentation. Therefore, solidstate fermentation of brown rice is a process that could enhance yield and overall production of enzymes and β-glucan for use in various applications.

## Introduction

Microorganisms play vital roles in the production of intracellular and extracellular enzymes on an industrial scale [1, 2]. The advantage of using microorganisms for the production of enzymes is that bulk production is economical; moreover, microbes can be easily manipulated to obtain enzymes with desired characteristics. In developing countries, studies regarding fungal enzymes (*e.g.*, amylase and protease) have focused mainly on *Aspergillus* species because of the ubiquitous nature and non-fastidious nutritional requirement of this organism [2, 3]. Amylases and proteases are universally produced throughout the animal, plant, and microbial kingdoms. The applications of these enzymes have increased throughout the clinical, medicinal, and analytical chemistry sectors. In addition, these enzymes are widely used in baking, brewing, manufacturing industries (*e.g.*, detergents, textiles, leather, and food), and agriculture [4, 5]. In this study, we employed the fungus *Aspergillus oryzae* to optimize the production of enzymes and β-glucan using solid-state fermentation (SSF).

Growth of hyphal tips is a method by which filamentous fungi form multicellular networks with branching cells at subapical regions [6]. Measurement of microbial activity or biomass is not straightforward under these conditions. Although not produced by all fungi, ergosterol concentrations are known to vary within each species depending on the physiological state and growth conditions of the fungus [7-10]. Ergosterol has both free and esterified forms in fungi. The free form is located in cell membranes while the esters are found in cytosolic lipid particles [11]. The ratio of these forms varies among different species [8], but total ergosterol concentration is needed for total fungal biomass quantification. Thus, total ergosterol concentration was used as a fungal biomarker for cell growth in SSF.

Brown rice (*Oryza sativa*) grains include bran, germ, and endosperm. They are generally covered with fine hairs and contain large amounts of nutrients such as soluble dietary fiber, proteins, fats, vitamins, minerals, and phytochemicals [12]. In addition, the endosperm cell walls of grasses typically have a high content of β-glucan, relative to the cell walls of non-graminaceous plants [13, 14]. To produce β-glucan, this study was conducted using brown rice as the source material in SSF.

SSF involves the growth of organisms on moist substrates in the absence of free-flowing water. The use of SSF for the production of enzymes and other metabolites offers many advantages over submerged fermentation, including greater economic viability, use of cheaper substrates, lower production costs, higher enzyme yields, and less energy consumption [15]. However, studies addressing enzyme production profile by SSF and its correlations with other biochemical changes (*e.g.*, β-glucan content) during fermentation are rare. The objective of this study was to evaluate the effects of the culture medium composition on enzyme production (*e.g.*, the concentrations of brown rice, rice bran, and ascorbic acid), as well as nitrogen source and concentration. SSF was conducted to demonstrate the feasibility of maximizing the production of enzymes and β-glucan from *A. oryzae*.

## Materials and Methods

### Microbial Strains and Culture Medium

*A. oryzae* KCCM 12698 was obtained from the Korean Culture Center of Microorganisms (Korea). The culture was propagated on potato dextrose agar (Oxoid) and stored at 4°C. A volume of 10 ml of 0.01% Tween 80 was added daily for 7 days to complete sporulation. Spores were scraped from the surface to prepare the spore suspension, which was then shaken for 2 h at 200 rpm (30°C) to produce a homogeneous spore suspension. The spores were counted using a hemocytometer; the inoculum comprised 1 × 10^8^ spores/ml [16]. One milliliter of spore suspension was inoculated into a 250-ml Erlenmeyer flask containing 100 ml of modified synthetic medium (30 g/l brown rice powder, 30 g/l rice bran, 10 g/l soytone, and 3 g/l ascorbic acid). The culture was incubated for 48 h at 150 rpm (30°C) on a rotary shaker and used as a seed culture. Ascorbic acid was added to the modified synthetic medium after sterile filtration with 0.22-μm filter pores. Nutrient supplements of 5 g/l K_2_HPO_4_ and 0.25 g/l MgSO_4_ were added to the modified synthetic medium and mixed thoroughly before inoculation.

### Evaluation of Synthetic Medium Conditions for Enzyme Production

One factor at a time experiments were carried out to optimize the main factors [17]. The composition of the synthetic medium used to culture *A. oryzae* KCCM 12698 was optimized as follows: brown rice concentration, 10–90 g/l; rice bran concentration, 0–90 g/l; nitrogen source and concentration, 5–60 g/l; and ascorbic acid concentration, 1–9 g/l. Various concentrations of brown rice were added to 250-ml Erlenmeyer flasks containing 100 ml of basal synthetic medium (20 g/l yeast extract, 50 g/l rice bran, and 1 g/l ascorbic acid). Under identical conditions, using a fixed brown rice concentration, nitrogen sources in the basal synthetic medium were replaced with various nitrogen sources (*e.g.*, 20 g/l of yeast extract, tryptone, beef extract, soytone, NH_4_Cl, and NH_4_NO_3_) to determine the suitable nitrogen source and optimal concentration for fungal biomass and enzyme production. The concentration of ascorbic acid was also optimized. Nutrient supplements of 5 g/l K_2_HPO_4_ and 0.25 g/l MgSO_4_ were added to the basal synthetic medium and mixed thoroughly before inoculation. Thus, production of amylase and protease was measured in liquid-state fermentation (LSF) of brown rice by altering individual synthetic medium conditions.

### Optimization of SSF

SSF was carried out at 30°C for 7 days using 500-ml stainless steel rectangular trays (CY-1070; 260 × 170 × 50 mm, Chunyangsa Co., Ltd., Korea) with a 200-ml working volume. Brown rice (Korea) was pretreated by soaking 100 g (of rice) in 200 ml water for 2 h in rectangular trays, which were then closed with aluminum foil, autoclaved at 121°C for 60 min, and cooled to room temperature. Nutrient supplements of fresh modified synthetic medium (10 ml) were added to the rectangular trays and mixed thoroughly before inoculation. Ten milliliters (0.0025 mg/g of ergosterol) of seed culture of *A. oryzae* KCCM 12698 was inoculated onto the surface of the autoclaved brown rice and mixed thoroughly under aseptic conditions. SSF was carried out at 30°C and 60%relative humidity using a Constant Temp & Humid Incubator (BH-120CA/B; Neuronfit Co., Ltd., Korea). Optimization of the fermentation parameters for SSF was carried out to determine the effect of each parameter on enzyme and β-glucan production. Samples were collected periodically, lyophilized using a freeze dryer (BFD85-F8; IlShinBioBase Co. Ltd., Korea), and stored at 20°C prior to measurements of amylase, protease, ergosterol, and β-glucan content.

### Determination of Fungal Biomass

During SSF, samples were collected periodically and lyophilized using a freeze dryer. Fungal growth was evaluated by determining the ergosterol yield in dry and homogenized biomass, using a modified version of the method described by Beni et al. [18]. Samples (1.0 g) were placed in 15-ml conical tubes, mixed with 5 ml of 0.07 M potassium hydroxide in methanol, and the tubes were tightly sealed with screw caps. Extraction was carried out in a shaking incubator for 1 h at 150 rpm (30°C). The tubes were subjected to ultrasonic shaking for 10 min, then centrifuged at 994 ×g for 5 min. The supernatant was collected, transferred to a new tube, and mixed with 150 μl of 0.1 M HCl. The sample extract was acidified to avoid ionization of ergosterol and to prevent degradation of the C-18 solid phase extraction (SPE) column. The supernatant solution was then passed through a C-18 SPE column using a Visiprep SPE vacuum Manifold (Merck KGaA, Germany). Ergosterols retained on the C-18 SPE column were eluted with 2 ml of isobutanol. The concentration of ergosterol was determined using high-performance liquid chromatography (Agilent 1200 Series; Agilent Technologies, USA) and a SUPERSIL ODS I-C18 column (250 × 4.6 mm). UV detection was performed using a photodiode array at a wavelength of 282 nm. The mobile phase consisted of 100% methanol with a flow rate of 1.0 ml/min at 30°C. Dilutions of standard solutions were used to compare and identify the obtained peaks.

### Enzyme Assays

Protease activity was determined using the method described by Qureshi et al. [19]. A 1-ml solution of protein substrate (10 g casein/l of 50 mM sodium phosphate buffer, pH 7.6) was mixed with 1 ml of the sample supernatant (5 g/100 ml) and incubated at 37°C for 10 min. The enzyme activity was then quenched by adding 2 ml of 0.4 M trichloroacetic acid. A 1-ml aliquot of this supernatant was mixed with 5 ml of 0.4 M sodium carbonate buffer (pH 7.5), then mixed with 1 ml of diluted Folin reagent. The mixture was held at room temperature for 30 min, after which optical density of the solution was measured in a spectrophotometer at 660 nm. The blank was prepared as above, but 2 ml of distilled water was used instead of the sample supernatant (1 ml) and the protein substrate (1 ml). One unit of protease activity was defined as the quantity of enzyme required to release 1 μmol tyrosine per minute from the substrate at 37°C.

Amylase activity was determined by measuring the reducing sugars released from soluble starch [19]. A 1.0-ml aliquot of the sample and an equal volume of the substrate solution (10 g of soluble starch per liter of aqueous solution) were mixed thoroughly and incubated in a water bath at 37°C for 15 min. The reaction was stopped by adding 2.0 ml of dinitrosalicylic acid reagent and boiled for 5 min in a boiling water bath. The solution was cooled under running water and spectrophotometric absorbance was measured at 540 nm against a blank. The blank was prepared as above, but the sample and substrate solutions were replaced with equal volumes of water. The reducing sugars released were measured using the dinitrosalicylic acid method [20]. One unit of amylase was defined as the amount of enzyme needed to liberate 1 μmol of glucose per minute from starch under the assay conditions specified above.

### Analysis of β-Glucan

The amount of β-glucan was analyzed using a mixed-linkage β-glucan assay kit supplied by Megazyme International. The activity of β-glucan was determined in accordance with the procedures described by Yoo et al.[21]. The β-glucan concentration was calculated using the following equation:

$$\beta -\text{glucan ( \%, w/w) = }\Delta \text{A }\times \frac{\text{F}}{\text{W}}\times \text{FV}\times \text{0.9}$$ (1)

Where ΔA is the absorbance after applying β-glucosidase to the reaction solution minus the absorbance of the reaction blank, F is a factor for converting absorbance values to the amount of glucose (μg), W is the weight of the extract analyzed (mg), and FV is the final volume (*i.e.*, 9.4 ml for rice flour). All analyses were conducted in triplicate, and the data were expressed as means ± standard deviations.

## Results and Discussion

### Profiles of Enzyme Activity and Fungal Biomass

**Fig. 1:**
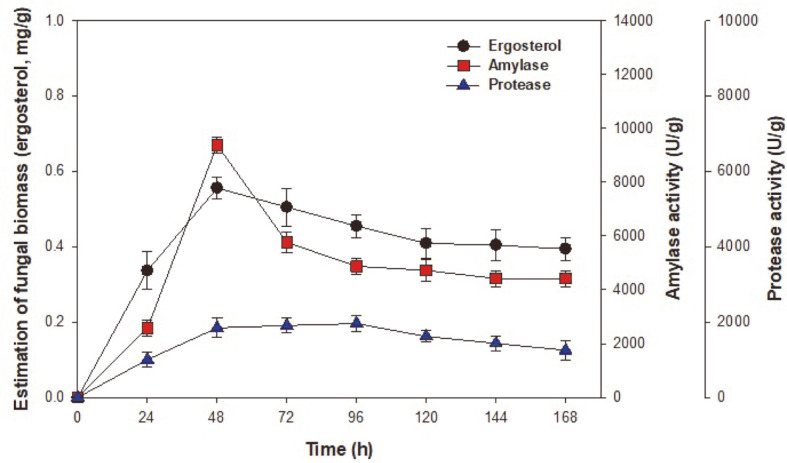
Time course profiles of fungal biomass (ergosterol) and enzyme production by *A. oryzae* KCCM 12698 using liquid-state fermentation in synthetic medium. The initial basic synthetic medium composition was 50 g/l brown rice, 50 g/l rice bran, 20 g/l yeast extract, 1 g/l ascorbic acid, and nutrient supplements. The initial pH was 5.5, the temperature was 30°C, and fermentation was conducted for 168 h.

Time course profiles of LSF subcultures were carried out with 100 ml of basal synthetic medium containing 50 g/l of brown rice, 20 g/l yeast extract, 50 g/l rice bran, and 5 g/l ascorbic acid in 250-ml Erlenmeyer flasks under anaerobic conditions for 168 h. As shown in Fig. 1, the growth of *A. oryzae* exhibited no lag time and entered a stationary phase after 48 h. The ergosterol concentration of fungal biomass initially increased to 0.56 mg/g, then decreased to 0.39 mg/g at the end of fermentation. Furthermore, during fermentation, the production of both amylase and protease increased progressively over time. The highest concentrations of amylase and protease produced were 9,384.82 U/g and 1,848.50 U/g, respectively, at 48 h. However, reductions in ergosterol concentration and enzyme production were observed at 72 to 168 h of LSF. The initial pH of the medium was 5.5, but decreased to pH 3.9 during fermentation (data not shown). These findings indicate that the pH of the synthetic medium is an important parameter for fungal biomass formation and enzyme production. Similar results were reported by Ellaiah et al. [22]; enzyme production strongly depended on the extracellular pH, which affected several metabolic and enzymatic processes, including the transport of various components across cell membranes, thereby influencing cell growth and metabolite production. LSF was carried out for 48 h to optimize enzyme production in this study.

### Effects of Brown Rice and Rice Bran on Fungal Biomass and Enzyme Production

**Fig. 2:**
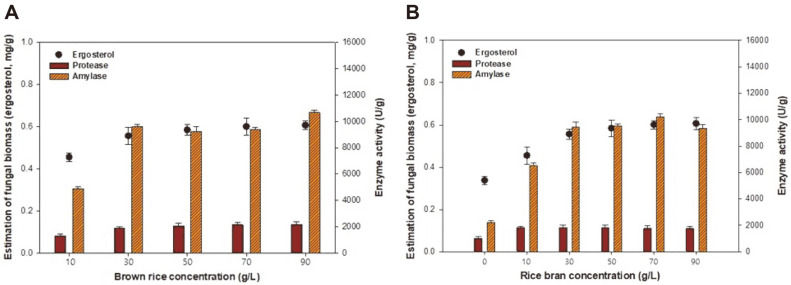
Effects of various concentrations of brown rice (A) and rice bran (B) on fungal biomass and enzyme production by *A. oryzae* KCCM 12698. The composition of synthetic medium was modified to include 30 g/l brown rice, 30 g/l rice bran, 20 g/l yeast extract, 1 g/l ascorbic acid, and nutrient supplements. The initial pH was 5.5, the temperature was 30°C, and fermentation was conducted for 168 h.

To optimize the production of amylase and protease in LSF subcultures, we modified the synthetic medium composition, focusing on each component individually. As shown in Fig. 2, the effects of brown rice concentration (Fig. 2A) and rice bran concentration (Fig. 2B) on enzyme production by *A. oryzae* KCCM 12698 were investigated. LSF was performed at 30°C and 150 rpm for 48 h, based on the results shown in Fig. 1.

Fig. 2A shows the fungal biomass and enzyme production of *A. oryzae* KCCM 12698 under different concentrations of brown rice, which was used as a carbon source. Our results indicated that the optimal concentration of brown rice was 30 g/l; further increases in concentration up to 90 g/l had no significant effects on fungal biomass and enzyme production. In addition, high brown rice concentrations led to high viscosity, which caused difficulty in handling the subculture and led to insufficient mixing, thereby reducing the efficiency of fermentation. Thus, a brown rice concentration of 30 g/l was selected as the optimal condition for enzyme production.

As shown in Fig. 2B, after the brown rice concentration had been fixed at 30 g/l, the basal synthetic medium was supplemented with various concentrations of rice bran. Both fungal biomass and enzyme production increased with rice bran concentration at 30 g/l. Fungal biomass, amylase, and protease concentrations reached 0.56 mg/g, 9,428.28 U/g, and 1,835.64 U/g, respectively. Further increases in rice bran concentration did not lead to significant increases in the levels of fungal biomass, amylase, or protease production. Compared with the control group (0 g/l), the addition of rice bran enhanced the fungal biomass and enzyme productivity in the LSF. These results suggest that the addition of rice bran has substantial potential for use in a cost-effective synthetic medium for the mass production of fungal biomass and enzymes [23]. Thus, 30 g/l of rice bran was selected as a suitable synthetic medium condition.

Notably, anticancer properties have been attributed to both brown rice and rice bran, following fermentation with *A. oryzae* [24]. For example, fermented rice bran acted as a preventive agent against colon carcinogenesis in rats. This suggests that brown rice and rice bran contain various health-promoting substances, including phenolic components with antioxidant activity. Phenolic components have a strong ability to inhibit the formation of reactive cell-damaging free radicals, or to scavenge existing radicals [25]. Accordingly, microbial fermentation of brown rice or rice bran is an emerging area of scientific and industrial research.

### Effects of Nitrogen Sources and Ascorbic Acid on Fungal Biomass and Enzyme Production

**Fig. 3:**
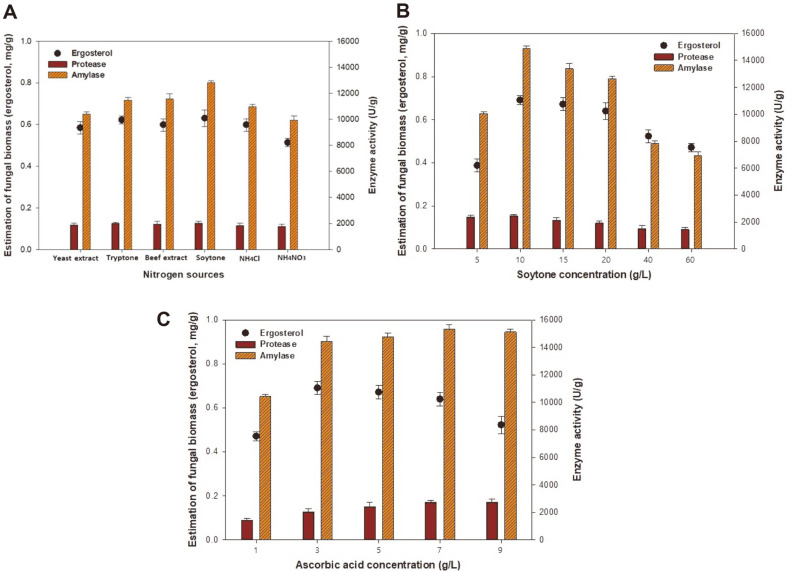
Effects of various nitrogen sources (A), soytone concentrations (B), and ascorbic acid concentrations (C) on fungal biomass and enzyme production by *A. oryzae* KCCM 12698. The modified synthetic medium composition was 30 g/l brown rice, 30 g/l rice bran, 20 g/l soytone, 3 g/l ascorbic acid, and nutrient supplements. The initial pH was 5.5, the temperature was 30°C, and fermentation was conducted for 168 h.

We evaluated various nitrogen sources and concentrations to enhance the enzyme production of *A. oryzae*. As shown in Fig. 3A, with soytone at 20 g/l, we obtained the highest levels of fungal biomass, amylase, and protease production at 0.63 mg/g, 12,956.29 U/g, and 1,931.30 U/g, respectively. Therefore, soytone was selected as the nitrogen source of choice in this study. There have been multiple studies regarding supplementation with nitrogen sources [26, 27]. A similar fermentation process was reported by Kammoun et al. [26] during an investigation of α-amylase production by *A. oryzae* CBS 819.72 grown on gruel (wheat grinding by-product). Organic nitrogen sources (*e.g.*, casein acid hydrolysate and soybean meal hydrolysate) have traditionally had a positive effect over inorganic sources because they are also a source of carbon; furthermore, they contain trace minerals and ions that could enhance enzyme secretion. However, the opposite effect was observed in *A. oryzae* [27]. None of the supplied organic nitrogen sources reportedly had any positive effect on enzyme production, although all sources promoted good fungal growth. As shown in Fig. 3B, the enzyme production of *A. oryzae* KCCM 12698 increased with increasing soytone concentration (up to 10 g/l), whereas enzyme production decreased as with increasing soytone concentration (from 15 to 60 g/l). The highest levels of fungal biomass, amylase, and protease production of 0.69 mg/g, 14,898.62 U/g, and 2,443.21 U/g, respectively, were obtained at a soytone concentration of 10 g/l. At concentrations that exceeded 10 g/l, the increase in production was almost negligible. Thus, 10 g/l soytone was selected as the preferred concentration for subsequent experiments.

As shown in Fig. 3C, the effect of the addition of ascorbic acid on enzyme production was assessed using concentrations of 1.0–9.0 g/l. At an ascorbic acid concentration of 3.0 g/l at 72 h, we obtained suitable levels of fungal biomass, amylase, and protease production of 0.69 mg/g, 14,431.03 U/g, and 2,043.76 U/g, respectively. Further increases in ascorbic acid concentration above 3.0 g/l resulted in a decrease in fungal biomass from 0.69 to 0.52 mg/g. However, the enzymatic activities of amylase and protease increased with increasing ascorbic acid concentration. Similar results were obtained by Yasui et al. [28], who suggested that vitamins were necessary for fatty acid synthesis and participated in the cellular energy supply. This may explain the increased enzyme activity that we observed with an increasing concentration of ascorbic acid. Therefore, for efficient enzyme production, 3.0 g/l ascorbic acid was selected as the optimal synthetic medium condition.

### Effect of Brown Rice Pretreatment Time on Enzyme and β-Glucan Production

**Fig. 4:**
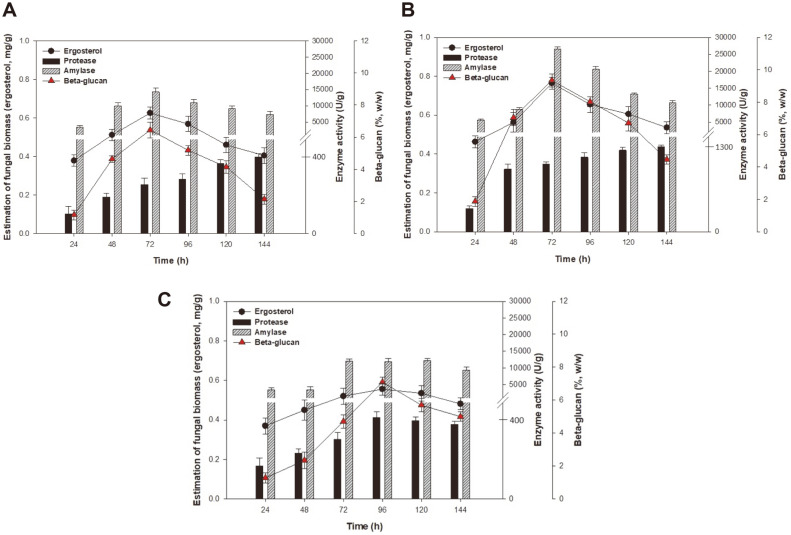
Evaluation of solid-state fermentation (SSF) under different pretreatment time conditions for brown rice: (A) 30 min, (B) 60 min, and (C) 120 min. SSF was carried out at 30°C and 50% relative humidity at 20°C.

Pretreatment of whole brown rice is important for mold growth and enzyme production in SSF. Water also plays an important role in the physicochemical properties of the substrate, thus affecting enzyme production [29]. Pretreatment for various durations of 30, 60, and 120 min was performed using 500-ml rectangular trays with a working volume of 200 ml. As shown in Fig. 4A, maximum levels of fungal biomass, amylase, protease, and β-glucan production at 0.62 mg/g, 14,237.06 U/g, 408.31 U/g, and 6.44% (w/w), respectively, were obtained when brown rice was pretreated for 30 min. When the pH increased to 7.0 at 96 h (data not shown), fungal biomass decreased, while the production of enzymes and β-glucan ceased. Similar results were obtained when soybean and wheat bran were used to enhance enzyme production from *A. oryzae* S [30]. An increase in pH (to 6.97) after 72 h of cultivation was reportedly due to the production of various extracellular proteins by *A. oryzae*. In our study, we also observed an increase in moisture levels with fermentation progression. Moisture levels changed from 50–60%to 70–80% after 144 h (data not shown). These results are in accordance with observations made by Ali et al. [31], who reported that moisture levels above 50% caused a decrease in enzyme production as the substrate became less porous, which adversely affected oxygen supply to the mold.

As shown in Fig. 4B, pretreatment of brown rice used in SSF for 60 min was carried out to further improve the production of fungal biomass, enzymes, and β-glucan. The maximum concentrations obtained were 0.76 mg/g, 26,551.03 U/g, 1,340.50 U/g, and 9.34% (w/w), respectively. Furthermore, the pH increased to 7.5 after 96 h of fermentation, and the moisture content changed from 40–50% to 50–60% during 144 h of fermentation (data not shown). These results indicate that 60 min of pretreatment in the SSF process had a positive effect on the formation of fungal biomass, enzymes, and β-glucan. This suggests that pretreatment for the approperiate duration could increase hydration homogeneity, degree of gelatinization, percentage of broken kernels, and degree of starch leaching, resulting in easy penetration of mycelia into the substrate (*i.e.*, brown rice) [32]. Thus, 60 min may be the optimal duration for substrate pretreatment in the SSF process.

As shown in Fig. 4C, with pretreatment of brown rice for 120 min, all products were slowly fermented and gradual increases in each of the products were observed with increasing fermentation time. Maximum levels of fungal biomass, enzymes (amylase and protease), and β-glucan production of 0.55 mg/g, 12,209.48 U/g, 421.79 U/g, and 7.09% (w/w), respectively, were obtained after 96 h of fermentation. Furthermore, the pH increased to 7.0 at 96 h of fermentation, and the moisture levels changed from 30–40% to 40–50% during 144 h of fermentation (data not shown). These results indicate that an extended pretreatment time could have a negative effect on the product yield due to the decrease in moisture levels and the formation of inferior brown rice texture. Similar results have been reported regarding inferior rice texture due to excessive heating [33]. The authors reported that under harsh conditions, such as extended hydrolysis time or high temperatures, rice texture was harder and stickier. Our findings indicate that the effective pretreatment time was 60 min in the SSF process.

Generally, oat- or barley-based β-glucan is tightly bound to starch, protein, and pentosan in the endosperm cell wall; thus, the extractability and characteristics of β-glucan vary depending on the source and extraction procedure. Yoo et al. [21] reported that the laboratory-scale extraction of β-glucan was 6.98 ± 1.17 g/100 g oat flour, which is similar to the β-glucan yield in our experiments. Thus, the SSF process allows efficient production of β-glucan, compared with the quantity obtained using extraction processes. These results indicate that SSF improves fungal biomass and enzyme production, while greatly improving β-glucan product yield from brown rice.

## Conclusion

In this study, *A. oryzae* KCCM 12698 produced a high fungal biomass and high concentrations of enzymes and β-glucan in an optimized synthetic medium under LSF. The composition of the synthetic medium was optimized using one factor at a time experiments. The ergosterol concentration determined by high-performance liquid chromatography at UV 254 nm was used as a biomass indicator for fungal growth. SSF of brown rice pretreated for 60 min at 121°C produced greater fungal biomass, as well as increased concentrations of enzymes and β-glucan. The developed SSF process comprises a simple and effective technique for the production of enzymes and β-glucan.
